# Nurse-led implementation of an insulin-infusion protocol in a general intensive care unit: improved glycaemic control with increased costs and risk of hypoglycaemia signals need for algorithm revision

**DOI:** 10.1186/1472-6955-7-1

**Published:** 2008-01-18

**Authors:** Kristin Alm-Kruse, Eva M Bull, Jon H Laake

**Affiliations:** 1 Department of Anaesthesia and Intensive Care Medicine, Rikshospitalet Medical Centre, 0027 Oslo, Norway

## Abstract

**Background:**

Strict glycaemic control (SGC) has become a contentious issue in modern intensive care. Physicians and nurses are concerned about the increased workload due to SGC as well as causing harm through hypoglycaemia. The objective of our study was to evaluate our existing degree of glycaemic control, and to implement SGC safely in our ICU through a nurse-led implementation of an algorithm for intensive insulin-therapy.

**Methods:**

The study took place in the adult general intensive care unit (11 beds) of a 44-bed department of intensive care at a tertiary care university hospital. All patients admitted during the 32 months of the study were enrolled. We retrospectively analysed all arterial blood glucose (BG) results from samples that were obtained over a period of 20 months prior to the implementation of SGC. We then introduced an algorithm for intensive insulin therapy; aiming for arterial blood-glucose at 4.4 – 6.1 mmol/L. Doctors and nurses were trained in the principles and potential benefits and risks of SGC. Consecutive statistical analyses of blood samples over a period of 12 months were used to assess performance, provide feedback and uncover incidences of hypoglycaemia.

**Results:**

Median BG level was 6.6 mmol/L (interquartile range 5.6 to 7.7 mmol/L) during the period prior to implementation of SGC (494 patients), and fell to 5.9 (IQR 5.1 to 7.0) mmol/L following introduction of the new algorithm (448 patients). The percentage of BG samples > 8 mmol/L was reduced from 19.2 % to 13.1 %. Before implementation of SGC, 33 % of samples were between 4.4 to 6.1 mmol/L and 12 patients (2.4 %) had one or more episodes of severe hypoglycaemia (< 2.2 mmol/L). Following implementation of SGC, 45.8 % of samples were between 4.4 to 6.1 mmol/L and 40 patients (8.9 %) had one or more episodes of severe hypoglycaemia. Of theses, ten patients died while still hospitalised (all causes).

**Conclusion:**

The retrospective part of the study indicated ample room for improvement. Through the implementation of SGC the fraction of samples within the new target range increased from 33% to 45.8%. There was also a significant increase in severe hypoglycaemic episodes. There continues to be potential for improved glycaemic control within our ICU. This might be achieved through an improved algorithm and continued efforts to increase nurses' confidence and skills in achieving SGC.

## Background

Hyperglycaemia, defined as blood glucose > 6.1 mmol/L [1], and insulin resistance are common in surgical and critically ill patients. These phenomena occur without previous diabetes, as a consequence of stress [1,2]. Elevated blood glucose is related to higher short and long-term morbidity and mortality rates, dependence on mechanical ventilation and hospital length of stay [1-5].

Previously, insulin-therapy in our ICU was utilised primarily when blood glucose was persistently above 12 mmol/L. However, the publication of papers by van den Berghe [1] describing reduced mortality, morbidity and length of stay in surgical patients subjected to intensive insulin-therapy, as well as by Finney [3], demonstrating an association of hyperglycaemia with adverse outcomes in general intensive care, led most doctors at our institution to prescribe insulin to our ICU-patients with a therapeutic target set at a BG level of 4 to 8 mmol/L. Equipped with a standard infusion solution of 2 IU/ml of rapidly acting insulin it was left to nurses to titrate treatment to achieve these targets. Without any protocol to guide treatment we became concerned that therapeutic targets were infrequently met and that both hyper- and hypoglycaemia might occur due to lack of commitment from both nurses and doctors.

van den Berghe and co-workers reported the results of a study of intensive insulin therapy in medical ICU-patients [6]. As in their previous study they found that patients who remained in the ICU for several days profited from SGC. Also, two studies from mixed ICU settings supported the notion that both medical and surgical patients may profit from improved glycaemic control [7,8]. These studies convinced us that we should both narrow our therapeutic target to that of normoglycaemia (4.4 to 6.1 mmol/L) and implement more rigid guidelines to achieve this goal.

SGC introduces a risk of severe hypoglycaemia (SHG) [1,9]. Pittas et al found that ICU-patients treated with insulin therapy had a threefold risk increase of developing hypoglycaemia compared with the control group. However, no adverse outcomes were reported after these incidents [2]. Another study explored the short-term consequences of SHG in the ICU. Out of 156 patients with SHG, there were three possible comas or seizures, and no increase in mortality [10]. On the other hand, in a case-control study Krinsley et al found severe hypoglycaemia to be independently associated with mortality in an adult ICU [11]. The incidence of SHG varies greatly in different studies with rates from 0.5 to 18.7 % [12]. Two projects led by nurses reported an 0.9 % incidence of SHG [13,14]. However, it is unclear whether these numbers refer to the percentage of blood glucose samples or of patients.

Another challenge for ICUs that would like to implement SGC is the increased workload for nurses: A blood glucose target of 4.4 to 6.1 mmol/L is a narrow corridor to operate within [13-16]. This will increase the need for monitoring and adjustment of insulin and nutrients. Thus, the motivation, acceptance, involvement and commitment of nurses are of importance for successful implementation of SGC [13-17]. It is known that to change the behaviour of a large group demands much more time and effort than simply passing on new knowledge. It is also important that those who are responsible are available for discussions and questions during implementation of new procedures [18]. Thus it was decided to assign joint leadership over this project to two ICU-nurses whose task it would be to develop and implement an algorithm for SGC in our ICU. A physician served as reference.

## Methods

### Ethics

The regional ethics committee waived the need for informed consent and classified our study as a "quality improvement" project. Permission for data collection was deferred to the hospital's data inspector who formally approved the study.

### Setting

Our hospital has a 44-bed department of mixed medical and surgical intensive care on a single floor. This study was limited to the 11-bed section of general intensive care for adults (the other three sections being paediatric, cardiothoracic surgical and general postoperative). The ICU attends to several national functions. The majority of patients in the section described are admitted after neurosurgery, abdominal- and bone marrow transplants, as well as with complications following acute cardiac conditions and other medical disease. The nurse: patient ratio is 1:1. The patients surveyed had a median age of 56 years, stayed for a median of 2.0 days, and were mechanically ventilated 77.6 % of the time.

Risk scoring was performed upon discharge or death using simplified acute physiology score II (SAPS II) with data obtained during the first 24 hours after admission. Only patients who either died in the ICU during the first 24 hours or had LOS > 24 hours were scored (Table 1).

**Table 1:** Patient charcteristics

	**2004–5**	**2006**
N	494	448
Age [IQR]*	56 [39–64]	55 [42–66]
Male Sex (%)	53.4	58.4
LOS [IQR]*	2.0 [0.7–6.4]	1.9 [0.8–5.8]
LOS > 72 h, n (%)	210 (42.5)	206 (46.0)
SAPS II [IQR]*	42 [28–54]	40 [30–54]
SAPS II completeness, n (%)^‡^	329 (67)	259 (58)

*) Age, LOS and SAPS II are given as median values [interquartile range].

^‡^) Frequency (%) of patients who received a SAPS II score. Eligible patients either died < 24 hours after admission or had LOS > 24 hours.

### Design

This was a combined retrospective analysis and prospective evaluation of glucose control in a single ICU with the purpose of implementing SGC in a safe manner.

In the retrospective part of the study (2004 – 2005) we mapped existing conditions regarding blood glucose regulation in our ICU. Because glycaemic control was already an issue, with BG limits set between 4–8 mmol/L, virtually all patients in the retrospective dataset received insulin (for data on insulin consumption, see "costs" in Results section). Data were retrieved from the laboratory database at the Department of clinical biochemistry. We analysed BG values from 15009 samples from 494 patients obtained during the 20 months before introduction of the algorithm, constituting a mean of 750 samples per month.

**Figure 1:**
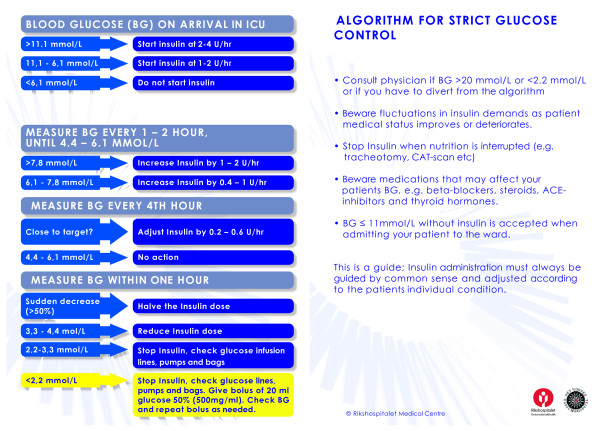
Glucose-infusion algorithm used in the study.

**Figure 2:**
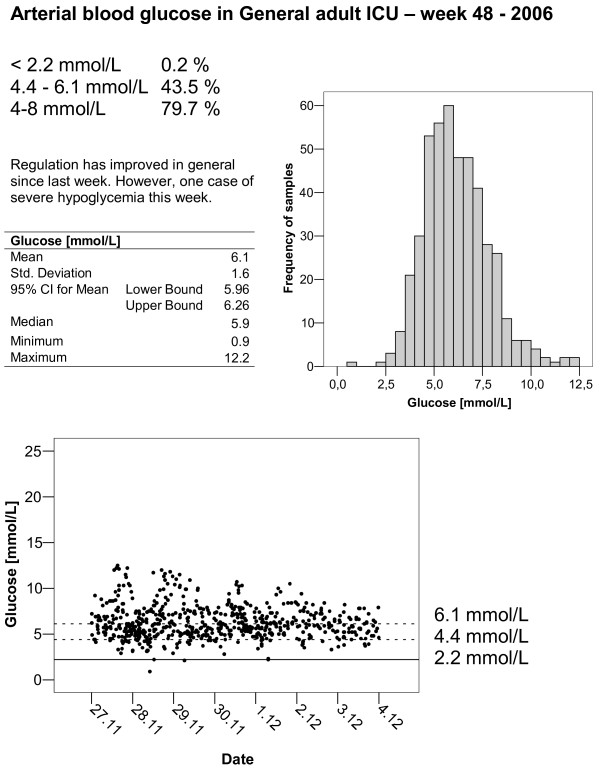
Example of feedback to ICU-nurses. Descriptive statistics were    presented on posters in the ICU and in e-mails to individual nurses.

The prospective study (2006) included six steps to safely implement SGC:

1. To obtain nurse commitment and a sense of "ownership" in the project, two nurses (KAK and EMB) were given daily leadership for the implementation of SGC. A physician (JHL) served as reference.

2. A blood glucose algorithm was developed (Fig 1). This was based on the Leuven- protocol [1], and adapted through discussions between ICU nurses and ICU physicians. Experiences from other ICUs in Norway were considered in the discussions. It was imperative that the algorithm should be easy to use and in pocketsize format. A graphic designer was consulted in designing the algorithm.

3. Lectures were provided for all physicians and nurses in the ICU. These lectures focussed both on original research on SGC, statistical results from our retrospective study and information about the algorithm.

4. Key literature was made easily accessible in the ICU.

5. Blood glucose statistics were analysed consecutively to assess performance and uncover incidences of hypoglycaemia. We analysed BG values from 24459 samples obtained from 448 patients over 12 months (2038 samples per month).

6. Feedback to nurses and doctors in the form of posters with simple descriptive statistics illustrated by coloured graphics were suspended on the main door to the rest area the ICU (Fig 2). Periodic summaries were e-mailed to the ICU staff. These analyses also functioned as a safety check for the project group as incidents of hypoglycaemia were quickly identified. The project nurses gave individual bedside feedback to the ICU nurses, and they were available for discussions and questions.

### Nutrition and insulin

Physicians in the ICU ordered nutrients and insulin per respective algorithms (see below). Glucose infusion was initiated at admission to ICU (normally 100 g per day in adults) and was usually discontinued when mixed enteral or parenteral nutrition commenced. Patients were fed according to our nutritional support algorithm [19]: This requires physicians to set a feeding target (normally 25–35 kcal/kg/day) and allows for use of standard mixtures of enteral and/or parenteral nutrition depending on gastrointestinal function (energy composition protein/amino acids ~15 %, fat/lipids ~35 %, carbohydrates/glucose ~50 %). With this algorithm 75% of our patients receives mixed nutritional support, including 150–300 g glucose, during the first day after admission, although full caloric support may not be achieved for several days [19].

Rapidly acting insulin (2 U/ml) was administered intravenously as a continuous infusion as described in the algorithm (Fig 1). The goal of the insulin treatment was to normalise high levels of BG as soon as possible or to maintain normoglycaemia. Virtually all patients received insulin. The hospital pharmacy provided information on yearly consumption of insulin by our ICU (see "costs" in Results section).

### Analysis

All patients admitted during the study period were included, regardless of length of stay and unless a physician specifically gave a written order that SGC was not to be initiated. The blood glucose measurements were arterial whole blood samples. We used four different blood gas analysers (Radiometer Copenhagen ABL 700) located in the ICU, which are regularly calibrated against a reference instrument at the Department of Clinical Biochemistry. The results of these analyses are automatically registered at the central laboratory database. Statistical analysis was performed with Statistical Package of Social Sciences (SPSS), version 13. For analysis of blood-glucose values we used both non-parametric statistics (Mann-Whitney's test) as well as "mixed model analysis" in which pre- and post implementation periods and length of stay in the ICU were treated as fixed factors and individual patients as random factors. Proportions were analysed with Chi-Square with Yates' correction.

### Costs

We calculated the incremental costs of insulin, an increased number of blood-gas analyses (syringes, chemicals), but excluding staff-expenditures (since manpower was not increased) as well as loss due to depreciation.

## Results

### Retrospective analysis (2004–5)

The retrospective analysis served as a reference to existing conditions, allowing us to evaluate the effect of the implementation of SGC. We obtained 15009 BG samples from 494 patients of whom 210 had a length of stay (LOS) in excess of 72 hours (Table 1). The median BG level was 6.6 mmol/L [IQR 5.6–7.7] (Table 2); 77.5 % of the BG measurements were within the then existing limits of 4–8 mmol/L and 33% of samples were within the "optimal" range of SGC, i.e. 4.4 to 6.1 mmol/L. Fourteen samples (0.1 %) from twelve patients (2.8 %) were defined as severely hypoglycaemic (< 2.2 mmol/L) (Table 3). Of these, two patients died while hospitalised at our institution and by 2007, 5 deaths were registered (all causes, Table 3). The median time interval from the first hypoglycaemic episode till death was 9 days. Hypoglycaemic patients had a median SAPS II score of 54 (mean score 55) while those hypoglycaemic patients who died had a median SAPS II score of 61 (mean score 60). We did not assess our patients for minor brain dysfunction after hypoglycaemia.

**Table 2:** Median arterial blood glucose

	**All samples** median (IQR)	≤**12 hours in ICU** median (IQR)	**> 12 hours in ICU** median (IQR)
Before algorithm (2004–5)	6.6 (5.6 – 7.7) n = 15009	7.1 (5.9 – 8.8) n = 1591	6.5 (5.6 – 7.6) n = 13418
After algorithm (2006)	5.9 (5.1 – 7.0) n = 24459	6.9 (5.4 – 8.6) n = 2928	5.9 (5.0 – 6.8) n = 21531

Median blood glucose values (IQR = interquartile range) of all samples obtained before and after implementation of an algorithm of strict glycaemic control. Differences between pre- and post algorithm values were significant for all subsets (Mann-Whitney's test). Mean blood glucose values in 2004–5 and 2006 were 6.82 and 6.25 mmol/L, respectively (mean difference 0.58 mmol/L, 95% confidence interval 0.54 to 0.62; p < 0.001; "mixed model analysis").

**Table 3:** Hypoglycaemia (arterial blood glucose < 2.2 mmol/L)

	**2004 – 5**	**2006**
Frequency of samples, n/N (%)^1^	14/15009 (0.09)	62/24459 (0.25)
Frequency of patients, n/N (%)^1^	12/494 (2.4)	40/448 (8.9)
Arterial blood glucose ^2^	1.8 mmol/L (1.3 – 2.1)	1.8 mmol/L (0.9 – 2.1)
30-day mortality (%) ^3^	3/12 (25)	12/40 (30)
Interval till death (days) ^4^	9 (0 – 533)	4 (0 – 53)
SAPS II score (deceased vs. survivors) ^5^	61 (39 – 86) vs 54 (26 – 75)	53 (31 – 96) vs 33 (15 – 60)

Footnotes:

1. P < 0.001 (Chi Square)

2. Median (range) of blood glucose values < 2.2 mmol/L

3. 30-day mortality among hypoglycaemic patients. In-hospital mortality was 2/12 (17%) vs. (10/40 (25 %), not significant.

4. Median number of days from first hypoglycaemic episode till time of death (range)

5. Median SAPS II score (range) of hypoglycaemic patients who died vs. patients who survived

### Introduction of strict glycaemic control (2006)

Following lectures, dissemination of literature and the new algorithm at the end of 2005, strict glycaemic control was swiftly introduced at the beginning of 2006 and met with little opposition. Only a handful of patients were deemed unsuitable for SGC by the attending physician (pancreas transplant recipients with their own BG regime), and our nurses enthusiastically embraced this therapy. Feedback regarding the algorithm was mainly positive. The nurses liked the design, and found it easy to understand and follow. The pocket size was appreciated. Inexperienced nurses tended to embrace it to a larger extent than experienced nurses. Nevertheless, some concern was raised about the algorithm, which did not prevent all hypoglycaemias (Table 3, see Additional file 1). This was evident especially where the BG level was declining rapidly, but due to the frequent measurements, fell outside the definition of 50 % decrease and was therefore not identified as a potential threat. In our experience, 4 hours between each sample when the previous test was within the normal limit, proved to be too long.

### Feedback and bedside follow-up

Posters with statistics and graphs received mixed interest. Due to technical reasons beyond our control the collection of data was in periods impeded, and this caused feedback to be less regular than we would have preferred. On the other hand periodic e-mails with similar content but also with comments on performance generated constructive discussions among the ICU staff as well as between the ICU staff and the project group. Also, bedside follow-up by the project nurses to guide colleagues in how to use the algorithm proved to be helpful.

### Glycaemic control

**Figure 3:**
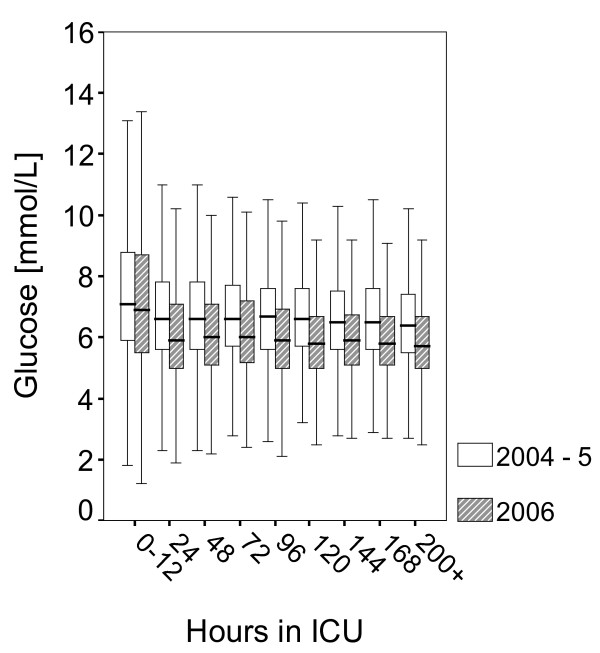
Boxplots represent median, quartiles, and extreme values of p-glucose samples [mmol/L] as a function of length of stay and year of admittance.

**Figure 4:**
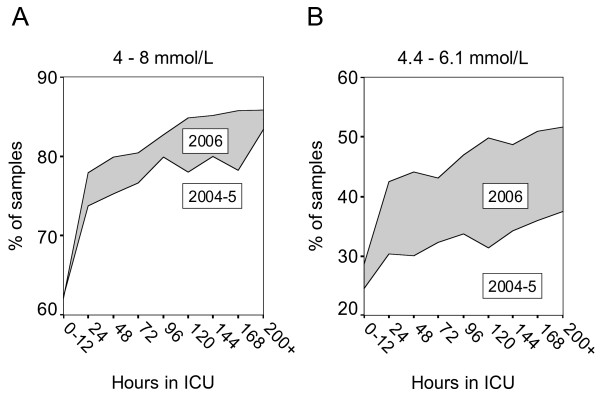
Percentage of samples with p-glucose between 4–8 mmol/L (A) and 4.4 – 6.1 mmol/L (B) as a function of length of stay and year of admittance.

**Figure 5:**
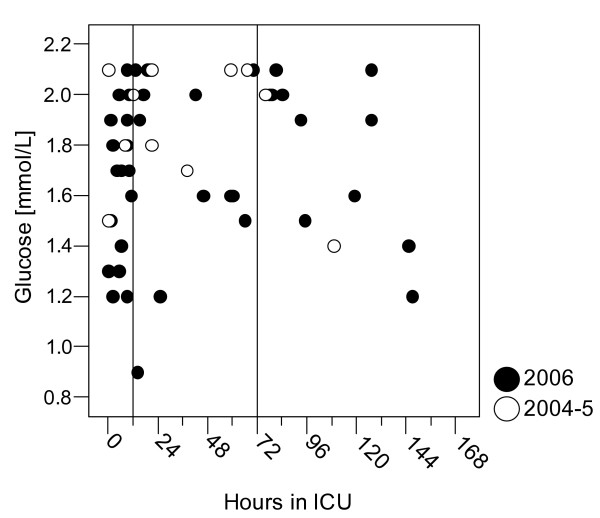
Hypoglycaemic episodes (BG < 2.2 mmol/L) plotted against length of stay. Reference lines are placed at 12 and 72 hours. Twenty-two episodes that occurred > one week after admission to ICU have been omitted from the figure.

Following introduction of SGC we obtained 24459 BG samples from 448 patients, of whom 206 had LOS > 72 hours (Table 1). The median BG level was 5.9 mmol/L [IQR 5.1–7.0]. Thus, the difference before and after implementation of SGC was 0.7 mmol and highly significant (p < 00.1, Table 2). Glycaemic control improved with length of stay in the ICU and was consistent after approximately 12 hours following ICU admission (Fig 3). We found that 80.9 % of the samples were within the previously defined limits of 4–8 mmol/L, while samples within the narrower range of 4.4–6.1 mmol/l had increased from 33 % in 2004–5 to 45.9 % in 2006, a relative increase of 39 % (Fig 4). The fraction of normoglycaemic samples improved with length of stay and the frequency of hyperglycaemic samples (> 8 mmol/L) fell from 19.2 % to 13.1 %, a relative decrease of 32 %. There was an increase in the incidence of severe hypoglycaemia; 40 patients (8.9 %) experienced one or more episodes of BG < 2.2 mmol/L (62 samples, 0.25 %). Of these, 10 patients died while hospitalised at our institution (25 %) and by 2007, 15 deaths were registered (all causes, Table 3, Additional file 1). The median time interval from the first hypoglycaemic episode till death was 4 days. Most hypoglycaemic incidents occurred during the first days after admission (Fig 5). Hypoglycaemic patients had a median SAPS II score of 39 (mean score 45) while those hypoglycaemic patients who died had a median SAPS II score of 53 (mean score 60). We did not assess our patients for minor brain dysfunction after hypoglycaemia.

Effect on mortality was not formally assessed. However, in 2004–5 and 2006 ICU-mortality was 17.5 % and 13.8 %, respectively (all admissions). For patients who remained in the ICU for more than 24 hours or died within the first 24 hours after admission, ICU- and 30-day mortality was 24.9 % and 32.6 % in 2004–5. In 2006 ICU- and 30-day mortality was 19.7 % and 28.6 %. The median SAPS II scores in 2005 and 2006 were 42 and 40, respectively (Table 1).

### Costs

The workload expanded from 750 blood-gas samples per month to 2038 samples per month. Insulin consumption was 303 000 IU per year in 2004, 379 000 IU per year in 2005 and 415 000 IU per year in 2006. This led to an increase in material costs (insulin, syringes, chemicals), from € 1561/month to € 3550/month, equating a 127 % rise in expenses, not considering personnel costs (since manpower was not increased) and loss due to depreciation.

## Discussion

### Responses to the introduction of SGC

Both education and the possibility to discuss experiences have been pointed out as success-criteria when implementing new protocols [13]. When teaching the ICU staff about the principles and practice of SGC, the main focus was on practicalities for the nurses and the benefits for the patients. During these sessions discussion was encouraged. We received few objections to the increased workload. The 1:1 nurse: patient ratio in our ICU may have contributed to the positive attitude. The staff already had experience in managing insulin infusions, albeit less strictly, when the BG target range was 4 to 8 mmol/L. The main reactions from both the nursing staff and physicians were interest, support and satisfaction. Such positive responses are described in the literature to be related to understanding the protocols rationale and benefits [13,20]. With very few exceptions doctors prescribed BG targets according to the new algorithm. From time to time it was necessary to curb enthusiasm from wards that wanted to participate in the study, and physicians who wanted to use the algorithm outside the ICU.

### Written feedback

Contrary to our expectations, posters with performance statistics generated little interest. On the other hand, e-mails with the same information caused discussions and proved valuable. It seems that e-mails allowed our co-workers to assess the feedback at their own pace and when it did not interfere with other obligations. We find this experience useful with respect to future quality enhancement projects.

### Glycaemic control

During 2004–5, 77.5 % of blood-glucose samples were within the target-range specified at that time, i.e. 4–8 mmol/L. The number of cases of severe hypoglycaemias was low. Thus, nurses were able to reach set targets even with no algorithm to guide therapy. The statistics indicate that nurses conservatively preferred to keep the BG levels in the higher end of the target range. This finding was confirmed in discussions with the nurses. The nurses' rationale was to avoid SHG. Several experienced nurses expressed uncertainty with the use of an algorithm to control the BG level instead of the 'good old way' of intuition and experience. However, following introduction of new therapeutic targets, an algorithm to guide therapy and extensive teaching and feedback, glycaemic control improved significantly, albeit at the cost of increased expenditure and more frequent hypoglycaemia. Was the reduction in median BG from 6.6 mmol/L to 5.9 mmol/L important enough to justify these risks? It is evident from Figs 3 and 4 that a downward shift of all BG values was the result of our project. Thus, the frequency of samples with BG > 8 mmol/L was reduced by 32 %. Finney et al found a strong association between higher level of BG and risk of death and suggested an upper tolerance limit of 8 mmol/L [3]. We are convinced that this can not be achieved unless one aims for a narrower range, i.e. normoglycaemia.

The studies by van den Berghe et al. [1,6] indicated that primarily patients who remained in the ICU for several days profited from SGC. This has resulted in suggestions that SGC be withheld until patients have remained in the ICU for three days. In our study 210 and 206 patients had LOS > 3 days in the periods before and after implementation of SGC, i.e. 44 %. Does this mean that the remaining patients were exposed to an unnecessary risk? Our interpretation of the results of the randomised trials is that any benefit of SGC accumulates with increasing LOS. As it is extremely difficult to predict LOS for patients who are admitted to the ICU, we believe it to be a sensible strategy to initiate SGC as early as possible. Routines must be developed, however, to avoid hypoglycaemia (see below).

Glycaemic control improved for the majority of our patients. These results may be partially explained by the bedside follow-up and continuous attention to the rationale for SGC by the project-nurses. Also, the 1:1 nurse: patient ratio ensured that nurses attended to only one patient at a time. Furthermore, almost all our ICU patients were fed using enteral nutrition, possibly another contributing factor to achieving SGC [17]. Our continued goal is that our nurses become comfortable with BG levels in the middle range of normal, thus avoiding hyperglycaemia that would otherwise occur because they are "playing it safe".

### Hypoglycaemia

The number of patients who experienced severe hypoglycaemia increased from 12 (2.4 %) to 40 (8.9 %) following implementation of SGC. This compares well with published results [1,6,12] but is nevertheless a cause for concern. It is difficult to establish causality, if any, between episodes of hypoglycaemia and subsequent deaths and to avoid bias we have consulted with independent expert who is now evaluating the cause of death in patients who were hypoglycaemic. The death rate among hypoglycaemic patients did not exceed expected mortality rate as assessed by SAPS II score. Also, patients who died after hypoglycaemia were at higher baseline risk of death than survivors (Table 3, Additional file 1). In a recent review, Cryer refers to laboratory work in non-human primates that demonstrated a need for prolonged (i.e. several hours) of severe hypoglycaemia (< 1.0 mmol/L) to reliably produce brain damage [21]. The situation may well be different in critically ill patients, in particular those whose metabolic demands are only marginally met due to their illness [11]. On the other hand, hyperglycaemia has consistently been shown to be associated with severity of brain damage in head injured patients or those with anoxic coma [22,23]. Kanji et al (2004) claims that to eliminate the incidence of hypoglycaemia completely is unrealistic since ICU patients have significant fluctuations in metabolic and endocrine demands [16].

Although mortality was not formally assessed, ICU- and 30-day mortality in 2005 and 2006 do not raise cause for alarm.

### Need for algorithm revision?

The hypoglycaemias that resulted from the introduction of our algorithm led to a suggestion that unless BG was truly stable, no more than two hours should pass between blood-glucose measurements. If uncertain, or with changes in the patients condition, nurses were encouraged to measure the BG even more frequent. Also, nurses were encouraged to compare their own assessment, based on experience, with the algorithm. Thus, the status of the algorithm changed from "binding" to "guiding". Most hypoglycaemic episodes occurred early after admission to our ICU (Fig 5). This may indicate that insufficient attention was given to the issues of nutrition and glycaemic control in the early phase when patients were being stabilised in the ICU. Also, our algorithm may be criticised because it contains some subjective language and therefore requires interpretation by experienced nurses. This could potentially represent a problem for new nurses in training. However, new nurses in our ICU always work together with an experienced coach and it is therefore unlikely that hypoglycaemia occurred as a result of errors made by inexperienced nurses.

On the other hand our algorithm is not dose defining and allows for some variability in how aggressively nurses may treat blood-glucose aberrations. A number of different protocols used achieve SGC were reviewed by Meijering et al who concluded that dynamic scale protocols, similar to ours, yielded the best results [17]. More recently published protocols are often dose defining and computer guided, and claim superiority over less rigorous protocols, such as ours, particularly with respect to avoiding hypoglycaemia. However, the numbers of patients included in many of these most recent studies are often small, typically ranging from twenty or less [14,24-27] to 50–60 patients [28-30] (but see below). In our study only a small fraction of blood samples were hypoglycaemic (0.25 %), but this nevertheless affected 8.9 % of patients following introduction of SGC (Table 3). This may indicate that a fairly large number of patients should be included before superiority of any particular protocol is claimed.

A good example of a dose defining insulin infusion protocol was recently published by Braithwaite et al. who reported the results in 24 trauma patients [31]. The protocol was arranged as a table with six columns containing detailed instructions on insulin infusion rates depending on BG. The patient was assigned a particular treatment column depending on his or her response to therapy. BG was controlled hourly until stable and thereafter every 2 hours. Target (blood-glucose < 6.1 mmol/L) was reached at a median time of 11 hours and BG thereafter fluctuated very little. The authors observed no cases of severe hypoglycaemia. If similar safety and efficacy can be demonstrated in a large patient sample this protocol is an attractive alternative to our algorithm, especially because it lends itself to computerization.

Thomas et al used a web-based insulin dose calculator to achieve tight glycaemic control in a 16-bed ICU [32]. A total of 601 patients were subjected to this protocol, which was amended after 502 patients to allow for a higher insulin dose. Only 19 episodes of severe hypoglycamia were noted throughout this implementation (3.1 %), but the frequency seemed to increase after the amendment. Also, Davidson and coworkers reported experiences with a computer-directed intravenous insulin system that resulted in rapid glycaemic control and a low incidence (2.6 %) of hypoglycaemia in a large patient cohort [33]. These authors contrast the use of a computer-directed system with that of van den Berghe [1], which depends on trained nurses. However, in a smaller study Shulman et al reported that computerised decision-supported intensive insulin therapy did not result in tight glycaemic control and that 10 % of patients became hypoglycaemic [30]. To us it seems only reasonable that nurse-commitment and skill will contribute to success in obtaining glycaemic control in the ICU, whether they are assisted by computerised protocols or not.

## Conclusion

A nurse-led implementation of an algorithm for strict glycaemic control was well received by the staff in our ICU. The data showed significant improvement in the regulation of BG in our patients, but there was an increase in cases of severe hypoglycaemia indicating a need for algorithm revision. We will continue to encourage nurses to feel confident in working to obtain normoglycaemia by focussing on the benefits and rationale of strict glycaemic control.

## Key messages

• To achieve strict glycaemic control in our ICU we selected two nurses to lead the implementation of a new insulin infusion algorithm

• All nurses in our ICU underwent training to obtain knowledge about the benefits and potential dangers of strict glycaemic control Nurses' confidence in using an insulin infusion algorithm was strengthened through daily feedback at the bedside and weekly statistics and graphs describing results

• This strategy led to a significant improvement in glycaemic control in our ICU, but also increased the number of patients who had an hypoglycaemic episode and highlighted a need for protocol revision

• Involving nurses in the implementation of new therapies results in commitment, confidence and a "sense of ownership" that improves performance

## List of abbreviations

BG – Arterial blood glucose

ICU – Intensive care unit

IQR – Interquartile range

LOS – Length of stay

SGC – Strict glycaemic control (arterial blood glucose 4.4 – 6.1 mmol/L)

SHG – Severe hypoglycaemia (arterial blood glucose < 2.2 mmol/L)

## Competing interests

The author(s) declare that they have no competing interests.

## Authors' contributions

KAK, EMB and JHL planned and designed the study, and analysed the retrospective data. KAK and EMB conducted the execution of the project (introduction of algorithm, follow-up and statistical feedback to nurses) and collected data during the prospective part of the study. KAK and JHL analysed the prospective data and wrote the paper. All authors have read and approved the final manuscript

## Pre-publication history

The pre-publication history for this paper can be accessed here:



## Supplementary Material

Additional file 1 Detailed description of patients with severe hypoglycaemia who ultimately died. The file contains clinical descriptions of those patients who suffered one or more incidences of severe hypoglycaemia and who ultimately died. Click here for file
