# Supplementary material for: Nurse-led implementation of an insulin-infusion protocol in a general intensive care unit: improved glycaemic control with increased costs and risk of hypoglycaemia signals need for algorithm revision
Source: BMC Nurs. 2008 Jan 18;7:1. doi: 10.1186/1472-6955-7-1 (PMC2245923; doi:10.1186/1472-6955-7-1)
Supplement: Additional file 1 — Detailed description of patients with severe hypoglycaemia who ultimately died. The file contains clinical descriptions of those patients who suffered one or more incidences of severe hypoglycaemia and who ultimately died. [file 1472-6955-7-1-S1.pdf]

Appendix

Detailed description of patients with severe hypoglycaemia who ultimately died

**2004-5**	**Reasons for admission to ICU**	**Specific therapy**	**Hypoglycaemia**	**Outcome**
Male 47 years	Aplastic anaemia<br>Sepsis<br>Left ventricular failure	Intraaortic balloon counterpulsation	1.5 mmol/L<br>Day of admission	Death on day of admission
Female 43 years	Fulminant liver failure with encephalopathy<br>Cerebral infarction<br>Esophageal haemorrhage<br>Multiple organ failure<br>Primary biliary cirrhosis	Sengstaken tube	1.3 mmol/L<br>9 days after admission	Death 9 days after admission
Female 63 years	Acute renal failure<br>Thalamic stroke<br>Pyoderma gangrenosum	Lower leg amputation	1.5 mmol/L<br>4 days after admission	Discharged from hospital. Death 544 days after admission
Female 66 years	Subarachnoid haemorrhage	Endovascular coil	2.1 mmol/L<br>1 day after admission	Discharged from hospital. Death 66 days after admission
Male 75 years	Sepsis<br>Subdural haematoma<br>Normal pressure hydrocephalus	Craniotomy<br>Ventriculoperitoneal shunt	2.1 mmol/L<br>2 days after admission	Moved to other hospital. Death 9 days after admission
**2006**	**Reasons for admission to ICU**	**Specific therapy**	**Hypoglycaemia**	**Outcome**
Male 46 years	Cardiac arrest<br>Dilated cardiomyopathy	Therapeutic hypothermia	1.2 mmol/L<br>1 day after admission	Moved to other hospital. Death 8 days after admission
Male 40 years	Hepatic encephalopathy<br>Hepatorenal syndrome<br>Severe respiratory distress<br>Hepatitis B	MARS<br>NO-inhalation	1.3 mmol/L<br>Day of admission	Death 2 days after admission
Female 52 years	Intestinal gangrene<br>Liver necrosis	Laparotomy	1.7 mmol/L<br>Day of admission	Death 1 day after admission
Male 55 years	Aspergillosis (lungs and liver)<br>GVHD<br>Myelofibrosis	Bone marrow tx	2.0 mmol/L<br>1 day after admission	Death 5 days after admission
Female 51 years	Intracranial hemorrhage<br>GCS = 3<br>Cerebral herniation on arrival	Organ procurement	1.4 mmol/L<br>Day of admission	Death 1 day after admission

Female 39 years	ARDS/Sepsis<br>Cardiac arrest<br>Intoxication	ECMO	1.6 mmol/L<br>Day of admission	Death 2 days after admission
Female 15 years	Severe cerebral infarction<br>Cerebral vasculitis<br>Moya Moya disease	High dose steroids	1.9 mmol/L<br>4 days after admission	Death 4 days after admission
Male 77 years	Peritonitis/sepsis<br>Anastomosis leakage<br>Rectal cancer	Laparotomy for anastomosis repair after rectosigmoidal resection	1.5 mmol/L<br>5 days after admission	Moved to other hospital. Death 25 days after admission
Male 48 years	Multiple organ failure<br>Myelodysplasia	Bone marrow tx	1.0 mmol/L<br>9 days after admission	Death 25 days after admission
Female 44 years	Massive subarachnoid haemorrhage with re-bleeding	Cerebroventricular drainage<br>Endovascular coil	1.4 mmol/L<br>6 days after admission	Death 9 days after admission
Female 59 years	Multiple organ failure<br>Alcoholic liver disease with encephalopathy<br>Toxic epidermal necrolysis	Immune globulin<br>Palliative care	1.9 mmol/L<br>Day of admission	Death 2 days after admision
Male 56 years	Respiratory failure<br>Bronchopleural fistula<br>Lung cancer	Left sided lobectomy	1.2 mmol/L<br>3 days after admission	Death 8 days after admission
Male 54 years	Sepsis<br>Multiple organ failure<br>Critical illness polyneuropathy<br>Dilated cardiomyopathy	Intraaortic balloon pump	1.9 mmol/L<br>65 days after admission	Death 69 days after admission
Female 54 years	Mediastinitis (aspergillosis)<br>Respiratory failure<br>Cystic Fibrosis	Bilateral pulmonary tx	1.8 mmol/L<br>20 days after admission	Death<br>38 days after admission
Female 20 years	Pulmonary aspergillosis<br>Autoimmune hepatitis	Liver tx	1.9 mmol/L<br>131 days after admission	Death 183 days after admission
